# Associative Learning of New Word Forms in a First Language (L1) and Haptic Referents in a Single-Day Experiment

**DOI:** 10.3390/ejihpe11020044

**Published:** 2021-06-21

**Authors:** Yutao Yang, Yan Yan, Misa Ando, Xinyi Liu, Toshimune Kambara

**Affiliations:** 1 Department of Psychology, Graduate School of Education, Hiroshima University, 1-1-1 Kagamiyama, Hiroshima 7398524, Japan; m206537@hiroshima-u.ac.jp (Y.Y.); m205403@hiroshima-u.ac.jp (Y.Y.); ryu191618@gmail.com (X.L.); 2 Program in Psychology, School of Education, Hiroshima University, 1-1-1 Kagamiyama, Hiroshima 7398524, Japan; m214658@hiroshima-u.ac.jp

**Keywords:** associative learning, dual coding theory, haptic features, new word forms, Japanese

## Abstract

This study focused on the associative learning of new word forms in the first language and haptic stimuli. In this study, healthy Japanese participants performed three-step tasks. First, participants made nine subjective evaluations of haptic stimuli using five-point semantic differential scales (e.g., regarding stickiness, scored from 1 (not sticky) to 5 (sticky)). Second, the participants carried out learning and recognition tasks for associative pairs of new (meaningless) word forms in their first language (Japanese) and haptic stimulus (H condition), and performed learning and recognition tasks for new (meaningless) word forms only (W condition). The order of conditions was counterbalanced among participants. Third, participants performed free recall tasks. The results of the recognition tasks showed that the proportions and response times of the W condition were better and faster, respectively, than those of the H condition. Furthermore, preference of haptic features negatively correlated with free recall scores of the H condition; however, there was no significant difference between the free recall scores of the H and W conditions. Our results suggest that new word forms were learned better than associative pairs of new word forms and haptic stimuli in a single day of learning. Furthermore, the free recall performance of word forms associated with haptic features could also be affected by their subjective evaluation (preference).

## 1. Introduction

Hellen Keller, during her early years, learned that every object and event in the world is associated with specific word forms, based on her associative learning of a new word form (e.g., “water”) and its corresponding haptic sensation [1]. Associative learning between stimuli can occur either consciously or unconsciously [2]. Words comprise associations between word forms and sensory-motor or emotional features [3,4]. Previous studies have found that associative learning of word forms and sensory-motor referents affect the recognition and recall processes of word forms [5,6,7,8,9,10,11,12,13,14,15,16,17,18]. For example, in a longitudinal study by Kambara and colleagues, it was reported that task performances of recognition tasks were better for an associative condition between unfamiliar word forms in a first language (Japanese) and visual features (meaningless figures) than another associative condition between unfamiliar word forms in the first language (Japanese) and auditory features (meaningless sounds) [12]. In addition, Liu and colleagues reported that pictorial referents promote recognition and retrieval processes of associative pairs of referents and new word forms in a second language (Chinese) for healthy native speakers of Japanese [14]. Previous neurobiological studies have also shown that the hippocampus plays an essential role in associating perceptual features, including written word forms [19,20,21]. In addition, a previous study showed that infants can learn associative pairs of word forms (pseudowords) and auditory referents (sounds) [22]. However, some previous studies have examined the multi-sensory connections between haptic and other senses [23,24,25]. These findings suggest that the learning and recognition processes of haptic features are similar to those of visual or auditory features [23,24,25]. For example, Assumpção and colleagues reported that the haptic features include contextual information for haptic search [23]. Pensky and colleagues reported that the recognition of visual features was better than that of haptic features [25]. In addition, a previous study investigated whether haptic features can affect the correct recognition of objects [26]. Liu and Song (2007) have shown that soft objects are easier to remember than hard objects [26]. Although these studies suggest that haptic features affect the recognition and perception of objects, there is no clear evidence of whether associative learning for new word forms and haptic stimuli affects the recognition and recall processes of word forms. 

In this study, we conducted a behavioral experiment to identify associative learning for new word forms and haptic features. The experiment consisted of four tasks. In the first one, by using five-point semantic differential scales, participants evaluated each haptic stimulus associated with haptic sensations (e.g., stickiness) [27] and emotional feelings consisting of familiarity, preference, and arousal [28,29]. In the second one, we asked the participants to learn associative pairs of new word forms and haptic referents (H condition) and word forms only (W condition) as learning tasks. In the third, after all learning tasks in each condition, participants performed a recognition task for each condition. In each recognition task, participants judged whether the presented word form was shown in the learning task. In the fourth one, participants wrote all word forms that they could freely recall. In this experiment, additional samples could not be collected after the onset of the COVID-19 pandemic for safety reasons. Therefore, this behavioral experiment may be considered as a pilot study of associative learning of new word forms and haptic referents for healthy human participants. Two predictions emerged from this experiment. The first predicted that participants would recognize the W condition more than the H condition. This hypothesis was supported by previous findings, which found that participants need more attentional resources for source memories than item memories [30]. If so, the attentional resources would affect the differences between the associative learning of new word forms (item memories) and haptic stimuli (source memories), and the learning of new word forms (item memories) only. The second predicted that subjective evaluations of haptic features would influence the task performances of the H condition in recognition and free recall tasks. Psycholinguistic research has shown that familiarity of the referents would affect the memory performances for the associations between word forms and visual referents [8].

## 2. Materials and Methods

### 2.1. Participants

Seventeen healthy university students (13 females; *M_age_* = 21.10; *SD_age_* = 1.98) participated in this study. The participants were native Japanese speakers and right-handed. Written informed consent was obtained from participants before the study. This study was approved by the ethical committee in the Graduate School of Education at Hiroshima University (code number: 2019089). This experiment was conducted maintaining the guidelines of the Declaration of Helsinki. Participants received a gift card (a QUO card) of 500 Japanese YEN (JPY) as monetary reward after the experiment.

### 2.2. Materials

We prepared 80 meaningless words (pseudowords) that include low meaningfulness and low associative values (shown in more detail below), and 20 haptic materials for this experiment (Tables S1 and S2). The meaningless words were selected from words which were examined in a Japanese psychological study (see Table S1) [31]. All meaningless words included two Japanese Katakana letters (e.g., *レミ* *in Japanese*, *remi*). Japanese Katakana is one of the main characters in the Japanese language [32]. The range of meaningfulness in selected words ranged from 30 to 79 [31]. On the other hand, the range of associative value in the words ranged from 45 to 74 [31]. Twenty haptic stimuli were collected from a home improvement store near the authors’ institution. These materials consisted of papers, carpets, and towels, among others (see Table S2).

Eighty meaningless words were separated into four word lists (A, B, C, and D word lists; see Table S1), with each word list including 20 meaningless words. We counterbalanced the A, B, C, and D word lists as word lists of the associative condition (H condition), word condition (W condition), and new word conditions (HN and WN conditions) in recognition tasks. In a group (*n* = 13), the H, W, HN, and WN conditions were C, A, D, and B word lists, respectively. In another group (*n* = 4), the H, W, HN, and WN conditions were D, B, C, and A word lists, respectively. The new word conditions (HN and WN conditions) were only used in recognition tasks, whereas associative condition (H condition) and word condition (W condition) were used in learning and recognition tasks.

### 2.3. Task Procedures

This behavioral experiment employed a within-subjects design. We used Superlab 4.5 and a Windows-based laptop (ProBook 650 G4) for word stimuli presentations and performance records during all tasks. The order of tasks was as follows: an evaluation task of haptic stimuli (e.g., sand paper), a learning task of the first condition, a recognition task of the first condition, a learning task of a second condition, a recognition task of the second condition, and a free recall task. The learning and recognition tasks of each condition were used as a pair. For instance, the learning task of H condition was immediately followed by the recognition task of H condition. We counterbalanced the order of conditions (H and W conditions) among the participants. The visual word stimuli presentation order was randomized in each task (the learning or recognition task) of each condition (H or W condition). The presentation order of haptic stimuli in the evaluation and learning tasks was fixed, since the experimenter correctly presented haptic stimuli to participants. By reversing the presentation order of haptic stimuli among participants, the presentation order of haptic stimuli in the evaluation and learning tasks was counterbalanced across the participants. Trials in H and W conditions were not randomly mixed during the learning and recognition tasks. Participants were given a break between the learning and recognition tasks of H and W conditions for resting and preparation for the next task. The break between each task was approximately one minute. 

**Figure 1:**
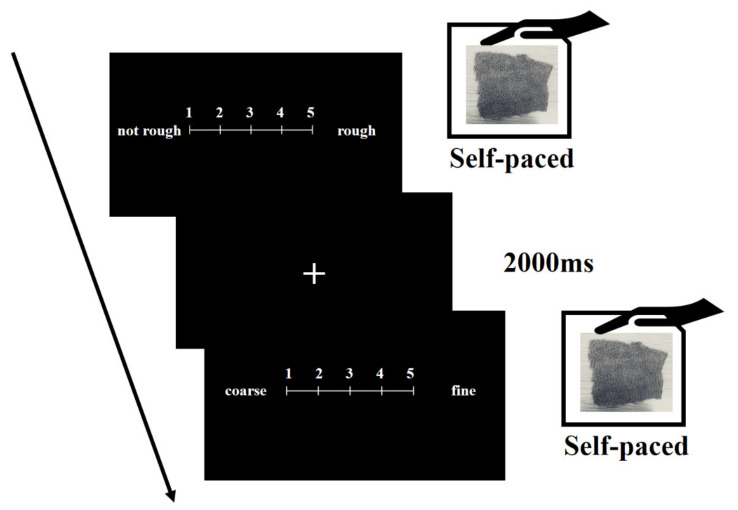
Evaluation task.

In the evaluation task of haptic stimuli, participants carried out subjective evaluations of presented haptic stimuli using five-point semantic differential scales [33] (see Figure 1). First, an experimenter (the first or second author) invisibly presented haptic stimuli to each participant’s left hand under a wooden enclosure in order to disturb the visual features of each haptic stimulus. Second, each participant touched each haptic stimulus. Third, each participant evaluated each stimulus by using the following 5-point semantic differential scales. Based on psychological findings [27,28,29,34,35,36], we decided to use the nine semantic differential scales associated with macro roughness (1 = not rough to 5 = rough), fine roughness (1 = coarse to 5 = fine), wetness (1 = dry to 5 = wet), hardness (1 = soft to 5 = hard), familiarity (1 = unfamiliar to 5 = familiar), warmness (1 = cold to 5 = warm), stickiness (1 = not sticky to 5 = sticky), preference (1 = hate to 5 = like), and arousal (1 = calm to 5 = excited). In the evaluation task, a fixation point (a cross mark) was presented for 2000 ms between trials for the participant to rest, decreasing the risk of mental and physical fatigue. Participants only looked at the fixation point during the presentation.

**Figure 2:**
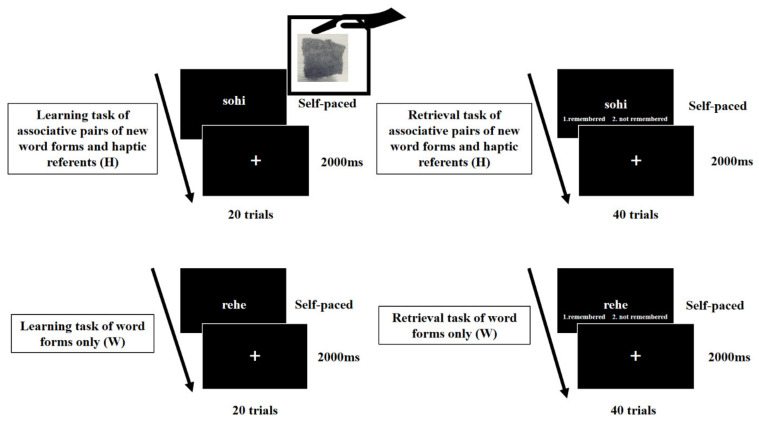
Learning and recognition tasks.

Thereafter, participants performed the learning and recognition tasks of the H and W conditions (see Figure 2). In the H condition, participants simultaneously learned 20 pairs of a new word form and haptic stimulus. The meaningless words were visually displayed on the laptop monitor, while the haptic stimuli were presented by the experimenter (the first or second author) to the participants’ left hand in a wooden enclosure for invisible presentation. The participants simultaneously memorized the new visually presented word form and the tactilely presented haptic stimulus (e.g., sand paper) as a referent of the visually presented word form. The duration of the stimulus presentation depended on key responses associated with each participant’s right index finger after learning each associative pair of new word forms and haptic stimuli. The key responses were measured as learning responses to associations between the word forms and haptic stimuli. Subsequently, the participants carried out the recognition task, where they judged whether the presented word was in the learning task using two keys (1: remembered; 2: not remembered). We used 20 words of the H condition and 20 meaningless words not presented in the learning task of the H condition (HN). Haptic feedbacks were not included in the recognition task for the H condition, since the control of the haptic feedback for each word form could not be conducted in the randomized presentation of the word forms. On the other hand, regarding the W condition, participants learned only 20 meaningless words in the learning task. The meaningless words were visually presented on the laptop monitor. Participants looked at and memorized each visually presented meaningless word in the learning task of the W condition. The duration of the stimulus presentation was dependent on key responses associated with each participant’s right index finger after learning each word form in order to record the key responses as the learning responses to the words. After the learning task of W condition, participants performed the recognition task of W condition that was the same as the recognition task of H condition. In the recognition task, we used 20 words of the W condition and 20 meaningless words not presented in the learning task of the W condition (WN). In both the learning and recognition tasks of H and W conditions, a fixation point (a cross mark) was presented between the trials for the participant to rest, to decrease the risk of mental and physical fatigue. While it was on the screen, participants only looked at the fixation point.

Finally, participants performed a free recall task, where the instruction was: “Please write all the words that you can remember.” The time limit was after the participants finished writing all the remembered word forms.

### 2.4. Analyses

We conducted four analyses. First, in order to identify differences in task performance between the H and W conditions, we conducted paired *t*-tests to determine proportions (0 to 1) and response times during the recognition tasks and proportions (0 to 1) during the free recall task. All the response times during the recognition tasks were with respect to hit trials (i.e., participants correctly judged presented word forms as learned word forms in the H or W conditions). Second, in order to exclude the effects of false alarm rates in each condition (i.e., participants incorrectly judged presented word forms as learned word forms in the HN or WN conditions), we calculated the corrected recognition scores (CRS) for each condition [37]. In the CRS calculation, we calculated the hit rate (i.e., each participant correctly judged presented word forms as learned word forms in the H or W conditions) minus the false alarm rate (i.e., each participant incorrectly judged presented word forms as learned word forms in the HN or WN conditions) for each participant. Although we did not use rates of miss (i.e., when participants incorrectly judged presented word forms as unlearned word forms in the H or W conditions) or correct rejection (i.e., when participants correctly judged presented word forms as unlearned word forms in the HN or WN conditions) in the CRS calculation, we examined the sum of hit and miss rates as well as false alarm and correct rejection rate, both of which were 1 for each participant. Subsequently, we compared the CRS of the H condition with that of the W condition. Third, we calculated each Cronbach’s alpha to examine the reliability of each semantic differential scale. Fourth, after the identification of reliable semantic differential scales, we conducted Pearson’s correlation analyses (or point-biserial correlations in cases of correlations between the task performances (i.e., proportions and response times of all trials) of the recognition and free recall tasks or correlations between task performances (i.e., proportions and response times of all trials) of the recognition and free recall tasks and subjective evaluations) among the reliable semantic differential scales and task performances (i.e., proportions and response times of all trials) of the recognition and free recall tasks. The proportions of the recognition and free recall tasks were coded as two values (0: miss; 1: hit). We conducted paired *t*-tests and correlation analyses using the software SPSS, and calculated Cohen’s d on a website (https://memory.psych.mun.ca/models/stats/effect_size.shtml (accessed on 20 June 2021)). In addition, since three participants did not perform the free recall task, we did not include their data in the analyses of free recall tasks and correlation analyses.

## 3. Results

### 3.1. Evaluation Tasks

We calculated the Cronbach’s alpha of each semantic differential scale to clarify reliability (Table 1). Cronbach’s alphas for macro roughness, fine roughness, wetness, hardness, familiarity, warmness, stickiness, preference, and arousal were α = 0.47, α = 0.52, α = 0.80, α = −0.13, α = 0.80, α = 0.46, α = 0.75, α = 0.73, and α = 0.84, respectively. In correlation analyses, we used five reliable semantic differential scales, including wetness, familiarity, stickiness, preference, and arousal, since the Cronbach’s alphas of these reliable semantic differential scales were higher than 0.70 [38].

### 3.2. Recognition Tasks

Regarding differences between proportions of the H and W conditions, we conducted a paired *t*-test, where we found that the proportions of the W condition were higher than those of the H condition (*t*(16) = 2.54, *p* < 0.05, *d* = 0.62; Table 2). In addition, based on previous findings [36], we conducted another paired *t*-test to compare the CRS of the H and W conditions. The results showed that the CRS of the W condition was better than that of the H condition (*t*(16) =3.94, *p* < 0.005, *d* = 0.95; Table 2).

Regarding differences between mean response times of hit trials in the H and W conditions, we also conducted a paired *t*-test and found that the mean response times of the W condition were faster than those of the H condition (*t*(16) = −2.98, *p* < 0.01, *d* = 0.72; Table 2).

### 3.3. Free Recall Task

The mean proportions of the H and W conditions in the free recall tasks were 0.16 (*SD* = 0.14) and 0.18 (*SD* = 0.18), respectively. The stimuli used in the experiment would affect the recall performances in both the H and W conditions (more details in Section 4). Moreover, we conducted a paired *t*-test of the comparison between mean proportions of the H and W conditions and found no significant difference (*t*(13) = 0.16, *p* = 0.88, *d* = 0.04; Table 3). 

### 3.4. Correlation Analyses

We found that a subjective evaluation of haptic features (preference) negatively correlated with the task performances of the H condition in the free recall task (*p* < 0.05). In addition, there were some significant correlations among the subjective evaluations of haptic features (see Table 4). First, stickiness was positively correlated with wetness (*p* < 0.01). Second, preference was positively correlated with familiarity (*ps* < 0.01), whereas preference was negatively correlated with arousal (*ps* < 0.01). Third, familiarity was negatively correlated with arousal (*p* < 0.05). Finally, the response times of all trials of the H condition in the recognition task negatively correlated with the task performances of the H condition in the recognition task (*p* < 0.01). 

## 4. Discussion

We investigated the differences in proportions and response times between the H and W conditions in the recognition and free recall tasks and found three main results. First, the proportions of the W condition were better than those of the H condition in the recognition tasks. Second, the response times of hit trials in the W condition were also faster than those in the H condition in the recognition tasks. Third, a subjective evaluation of haptic stimuli (preference) negatively correlated with the task performances of the H condition in the free recall task. These findings suggest that new word forms were better learned than associative pairs of new word forms and haptic stimuli in a single day. Furthermore, the free recall performance of word forms associated with haptic features could also be affected by subjective evaluation (preference) of the haptic features.

### 4.1. Recognition and Free Recall Performances

The results of recognition tasks showed that the recognition proportions and response times of the W condition were significantly higher and faster, respectively, than those of the H condition. These findings suggest that the recognition performances of word forms only were better than those of associative pairs of new word forms and haptic features. These findings are also consistent with findings in other studies. Word forms only were recognized better than associative pairs of word forms and pictures [14,17] or tastes [18] in a single-day learning. Since more attentional resources would be required for sources (referents or meanings) than for items (word forms) in memory tasks [30], current and other findings would show that word forms only could be recognized more than associative pairs of word forms and perceptual referents in a single-day learning.

In the free recall task, the mean proportions of the H and W conditions were lower than 0.20. The low proportions would be associated with the meaningless words (pseudowords) used in this experiment. In general, the recall performances of real words are better than those of pseudowords [39]. Additionally, the participants in this study only learned the pseudowords once in learning tasks. Thus, the pseudowords and learning time could influence the low free recall performances in this study.

### 4.2. Relationships between Preference of Haptic Stimuli and Free Recall Performance Associations

We found that the preference of haptic stimuli negatively correlated with the proportions of free recall tasks. This finding suggests that subjectively emotional features of haptic stimuli could decrease the recall performances of associative pairs of word forms and haptic referents. This finding was supported by previous findings. Associative pairs of visual features were learned better with neutral emotion than with negative emotion [40]. The emotional features of stimuli decrease the memory performances of associations between stimuli [41,42,43,44]. Li and Wang (2020) also showed that positive emotion decreases the memory performances of associative pairs of word forms and referents (e.g., definitions or pictures) [45]. Taken together with the previous and current findings, emotional features could decrease the memory performances for associations between word forms and perceptual referents.

### 4.3. Future Directions

As a first step, we conducted this behavioral experiment in a single day, since we firstly examined associative learning of new word forms and haptic references. Although in single-day learning, participants can effectively learn associations between word forms and perceptual features [14,17,18], associative learning of new word forms and visual or auditory stimuli for more than two days could increase the recognition and recall performances of the learned words, compared to the single-day learning [8,12,16]. If the experimental period can be expanded to more than two days, we may detect more effective and detailed associative processes for new word forms and haptic stimuli.

Additionally, although we examined relationships between the associative learning of new word forms and haptic features as well as the subjective evaluations of haptic features, working memory would also affect the associative learning of new word forms and haptic features. In fact, the repetition of pseudowords or verbal sounds is associated with associative learning of word forms and perceptual features [17], or language comprehension [46,47]. In future, we may need to examine relationships between associative learning of new word forms and haptic features and working memory.

## Figures and Tables

**Table 1:** Descriptive statistics of each semantic differential scales.

Semantic Differential Scales (Item Means)	Cronbach’s Alpha	*M*	MIN	MAX	Range	MAX/MIN	Variance	Number of Items
macro roughness	0.47	2.74	1.82	3.41	1.59	1.87	0.24	20
fine roughness	0.52	2.97	2.47	3.88	1.41	1.57	0.17	20
wetness	0.80	1.18	1.06	1.29	0.24	1.22	0.01	20
hardness	−0.13	3.16	2.35	4.00	1.65	1.70	0.25	20
familiarity	0.80	2.86	2.24	3.65	1.41	1.63	0.20	20
warmness	0.46	2.94	2.59	3.41	0.82	1.32	0.07	20
stickiness	0.75	1.17	1.06	1.41	0.35	1.33	0.01	20
preference	0.73	3.06	2.47	3.59	1.12	1.45	0.12	20
arousal	0.84	2.22	1.76	2.71	0.94	1.53	0.08	20

*M*: mean; MIN: minimum value; MAX: maximum value. These values were calculated on SPSS.

**Table 2:** Descriptive statistics of proportions and response times of the associative condition of new word forms and haptic stimuli (H condition), and the condition of new word forms only (W condition) in recognition tasks.

	H Condition *M* (*SD*)	W Condition *M* (*SD*)	*t*	*p*	*d*
HIT	0.70 (0.14)	0.78 (0.16)	2.54	0.02	0.62
FA	0.21 (0.11)	0.15 (0.11)	-	-	-
CRS	0.49 (0.17)	0.63 (0.19)	3.94	0.001	0.95
RT	3516.46 (396.36)	3336.41 (282.07)	−2.98	0.009	0.72

HIT: hit rate; FA: false alarm rate; CRS: corrected recognition score [37]; RT: mean response time of hit trials; *M*: mean proportion; *SD*: standard deviation; *t*: *t*-value; *p*: *p*-value; *d*: Cohen’s *d*. In the CRS calculation, we calculated the hit rate (i.e., participants correctly judged presented word forms as learned word forms in the H or W conditions) minus the false alarm rate (i.e., participants incorrectly judged the presented word forms as learned word forms in the HN or WN conditions) for each participant. The paired *t*-tests were conducted on SPSS.

**Table 3:** Descriptive statistics of proportions of the associative condition of new word forms and haptic stimuli (H condition), and the condition of new word forms only (W condition) in the free recall task.

H Condition *M* (*SD*)	W Condition *M* (*SD*)	*t*	*p*	*d*
0.16 (0.14)	0.18 (0.18)	−0.15	0.88	0.04

*M*: mean proportion; *SD*: standard deviation; *t*: *t*-value; *p*: *p*-value; *d*: Cohen’s *d*. The paired *t*-test was conducted on SPSS.

**Table 4:** Results of correlation analyses (*n* = 14).

	PR	PFR	RTR	S	W	P	F	A
PR	1							
PFR	0.06 ^+^	1						
RTR	−0.21 **^+^	0.09 ^+^	1					
S	0.04 ^+^	−0.04 ^+^	−0.07	1				
W	0.06 ^+^	−0.05 ^+^	−0.06	0.63 **	1			
P	0.03 ^+^	−0.13 *^+^	0.05	0.01	0.11	1		
F	−0.01 ^+^	−0.10 ^+^	−0.06	−0.05	−0.03	0.56 **	1	
A	−0.03 ^+^	0.03 ^+^	−0.04	0.03	−0.05	−0.42 **	−0.15 *	1

PR: task performances of recognition task; PFR: task performances of free recall task; RTR: response times of all trials in recognition task; S: stickiness; W: wetness; P: preference; F: familiarity; A: arousal; **: *p* < 0.01; *: *p* < 0.05. PR and/or PFR were coded as two values (i.e., 0: hit; 1: miss). +: We conducted point-biserial correlation analyses among PR, PFR, RTR, and subjective evaluations of haptic stimuli. Additionally, we conducted Pearson’s correlation analyses among the variables, excluding PR and PFR.

## Data Availability

The analyzed data are available on request to the corresponding author.

## Supplementary Materials

The following are available online at https://www.mdpi.com/article/10.3390/ejihpe11020044/s1, Table S1: Japanese word stimuli (transliteration), Table S2: Haptic stimuli.
