# Supplementary material for: Associative Learning of New Word Forms in a First Language (L1) and Haptic Referents in a Single-Day Experiment
Source: Eur J Investig Health Psychol Educ. 2021 Jun 21;11(2):616–26. doi: 10.3390/ejihpe11020044 (PMC8314365; doi:10.3390/ejihpe11020044)
Supplement: Supplementary file 1 [file ejihpe-11-00044-s001.zip › ejihpe-1235288-supplementary.pdf]

Table S1. Japanese word stimuli (transliteration).

**Word list A**	**Word list B**	**Word list C**	**Word list D**
レヘ (rehe)	ヌナ (nuna)	ソヒ (sohi)	ヌハ (nuha)
ネメ (neme)	ムヘ (muhe)	ヘメ (heme)	ラエ (rae)
ルユ (ruyu)	ワノ (wano)	ロヌ (ronu)	ツア (tsua)
ツソ (tsuso)	ヌツ (nutsu)	ヌサ (nusa)	ヌモ (numo)
ネワ (newa)	ホヌ (honu)	ヘハ (heha)	ムマ (muma)
メナ (mena)	ユチ (yuchi)	メミ (memi)	ラモ (ramo)
リイ (rii)	ルヤ (ruya)	ルニ (runi)	レチ (rechi)
レネ (rene)	ワホ (waho)	ロヘ (rohe)	ケヘ (kehe)
スヨ (suyo)	テセ (tese)	セホ (seho)	トヌ (tonu)
ニミ (nimi)	ヌヘ (nuhe)	ヌソ (nuso)	ヌワ (nuwa)
ネノ (neno)	ヘク (heku)	ヒセ (hise)	ヘフ (hefu)
ホミ (homi)	ムア (mua)	ミヒ (mihi)	ムト (muto)
ムル (muru)	ヨヤ (yoya)	ユヌ (yunu)	ラヤ (raya)
ラル (raru)	リフ (rifu)	リテ (rite)	ルセ (ruse)
ルメ (rume)	レハ (reha)	ルワ (ruwa)	ロナ (rona)
ロヨ (royo)	エニ (eni)	ワモ (wamo)	オヌ (onu)
サヘ (sahe)	スヘ (suhe)	スニ (suni)	セサ (sesa)
セモ (semo)	チテ (chite)	ソユ (soyu)	チネ (chine)
ツサ (tsusa)	ニヘ (nihe)	トエ (toe)	ヌア (nua)
ヌホ (nuho)	ネシ (neshi)	ヌユ (nuyu)	ノヒ (nohi)

Table S2. Haptic stimuli.

number	material	picture	number	material	picture	number	material	picture	number	material	picture
1	Woolen blanket	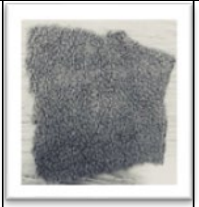	6	Gauze	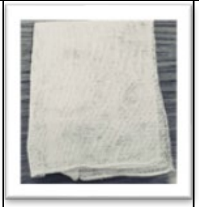	11	Foam board	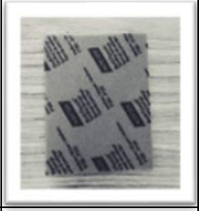	16	Hard gauze	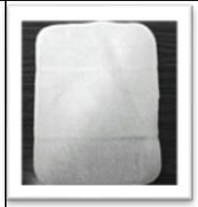
2	Soft mat	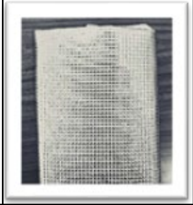	7	Fabric	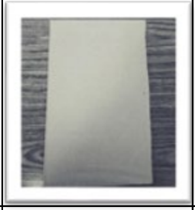	12	Plastic cutting board	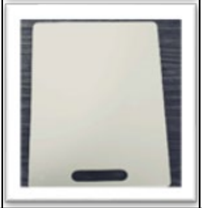	17	Towel	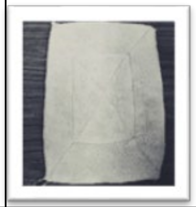
3	Wooden cutting board	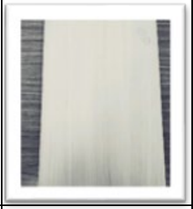	8	Sand paper	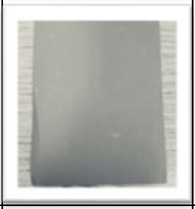	13	Sand paper	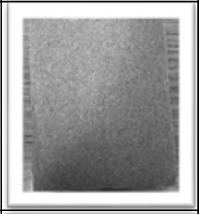	18	Hard mat	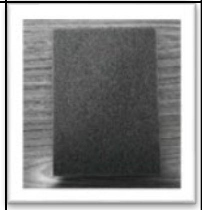
4	Foam board	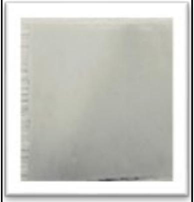	9	Rubber	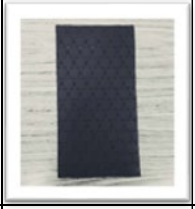	14	Hard mat	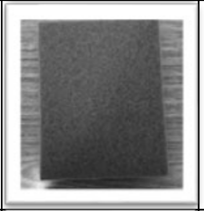	19	Vinyl sheet flooring	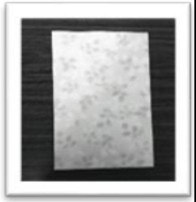
5	Foam board	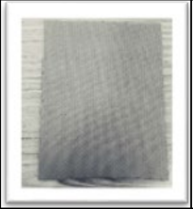	10	Artificial turf	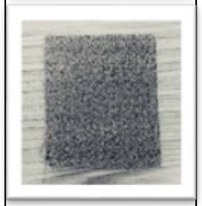	15	Filtration fabric	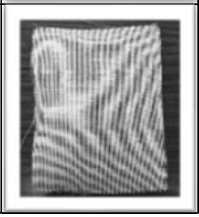	20	Aluminum	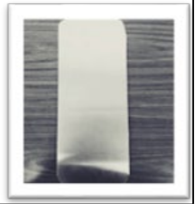

Note: Although same category names are included in Table S2, the materials of haptic stimuli are differential among those category names.
